# Characteristics and Disease Severity of US Children and Adolescents Diagnosed With COVID-19

**DOI:** 10.1001/jamanetworkopen.2021.5298

**Published:** 2021-04-09

**Authors:** Leigh Ellyn Preston, Jennifer R. Chevinsky, Lyudmyla Kompaniyets, Amy M. Lavery, Anne Kimball, Tegan K. Boehmer, Alyson B. Goodman

**Affiliations:** 1 COVID-19 Response Team, Centers for Disease Control and Prevention, Atlanta, Georgia; 2 Epidemic Intelligence Service, Center for Surveillance, Epidemiology, and Laboratory Services, Centers for Disease Control and Prevention, Atlanta, Georgia; 3 Commissioned Corps, US Public Health Service, Rockville, Maryland

## Abstract

This cohort study uses data from the Premier Healthcare Database Special COVID-19 Release to assess the association of demographic and clinical characteristics with severe COVID-19 illness among hospitalized US pediatric patients with COVID-19.

## Introduction

In 2020, more than 2 000 000 pediatric COVID-19 cases were reported in the United States.^1^ Although approximately half of pediatric patients with COVID-19 experience mild disease, some children require admission to intensive care units or use of invasive mechanical ventilation.^2^ We conducted a cohort study to estimate adjusted associations between demographic and clinical characteristics and severe COVID-19 among hospitalized pediatric patients.

## Methods

Discharge data from 869 medical facilities that contributed inpatient and emergency department encounters to the Premier Healthcare Database Special COVID-19 Release (PHD-SR) (release date, December 9, 2020), an administrative all-payer database capturing approximately 20% of US hospitalizations,^3^ were used to describe patients 18 years or younger who had an inpatient or emergency department encounter with a primary or secondary COVID-19 discharge diagnosis from March 1 through October 31, 2020. The *International Statistical Classification of Diseases, Tenth Revision, Clinical Modification* (*ICD-10-CM*) diagnosis code U07.1 was used from April 1 through October 31, 2020, and code B97.29 was used from March 1 through April 30, 2020. The discharge data were also used to estimate associations between demographic and clinical characteristics and severe COVID-19 among pediatric patients hospitalized with COVID-19. This study was reviewed by the Centers for Disease Control and Prevention and was deemed exempt from institutional review board oversight per 45 CFR §46.101(b)(4) and exempt from patient informed consent based on 45 CFR §164.506(d)(2)(ii)(B) because the disclosed PHD-SR data are considered deidentified. This study followed the Strengthening the Reporting of Observational Studies in Epidemiology (STROBE) reporting guideline.

Severe COVID-19 was defined as care requiring treatment in an intensive care unit or step-down unit, involving use of invasive mechanical ventilation, or resulting in death. We used the Agency for Healthcare Research and Quality Chronic Condition Indicator tool^4^ to identify chronic conditions using *ICD-10-CM* diagnoses from January 1, 2019, up to and including the child’s initial COVID-19 encounter. Race/ethnicity was defined by information in patient medical records in the PHD-SR.

Adjusted odds ratios (AORs) and 95% CIs for severe COVID-19 were calculated using a multivariable generalized estimating equations model adjusting for within-facility correlation, age, sex, race/ethnicity, insurance type, and presence of chronic conditions. Statistical analysis was performed using SAS version 9.4 (SAS Institute Inc). The a priori significance level was set at *P* = .05; all hypothesis testing was 2-sided.

## Results

Among 20 714 pediatric patients with COVID-19, 10 950 (52.9%) were girls, 11 153 (53.8%) were aged 12 to 18 years, 8148 (39.3%) were Hispanic or Latino individuals, 5054 (24.4%) were non-Hispanic Black individuals. Among these patients with COVID-19, 6047 (29.2%) had 1 or more chronic conditions (Table).

**Table.** Demographic and Clinical Characteristics, Care Setting, and Severity of Patients 18 Years or Younger With COVID-19, United States, March to October 2020^a^

Characteristic	No. (%)					
	Total pediatric cohort in PHD-SR^b^	Pediatric COVID-19 cohort	Hospitalized with COVID-19	Hospitalized with severe COVID-19^c^	ICU admission	Invasive mechanical ventilation
Total, No.	1 945 831	20 714	2430	756	747	172
Portion of pediatric COVID-19 cohort, %	NA	100	11.7	3.6	3.6	0.8
Sex						
Female	966 861 (49.7)	10 950 (52.8)	1344 (55.3)	352 (46.6)	345 (46.2)	72 (41.9)
Male	976 884 (50.2)	9742 (47.0)	1083 (44.6)	404 (53.4)	402 (53.8)	100 (58.1)
Age, y						
0-1	726 354 (37.3)	3606 (17.4)	632 (26.0)	153 (20.2)	152 (20.4)	43 (25.0)
2-5	305 500 (15.7)	2458 (11.9)	245 (10.1)	104 (13.8)	103 (13.8)	22 (12.8)
6-11	327 429 (16.8)	3497 (16.9)	274 (11.3)	119 (15.7)	119 (15.9)	20 (11.6)
12-18	586 548 (30.1)	11 153 (53.8)	1279 (52.6)	380 (50.3)	373 (49.9)	87 (50.6)
Race/ethnicity^d^						
Non-Hispanic or Latino						
White	935 117 (48.1)	5083 (24.5)	639 (26.3)	194 (25.7)	191 (25.6)	38 (22.1)
Black	358 139 (18.4)	5054 (24.4)	478 (19.7)	184 (24.3)	182 (24.4)	50 (29.1)
Asian	43 897 (2.3)	396 (1.9)	56 (2.3)	21 (2.8)	21 (2.8)	Suppressed^e^
Other	149 797 (7.7)	1370 (6.6)	191 (7.9)	59 (7.8)	59 (7.9)	Suppressed^e^
Hispanic or Latino	364 012 (18.7)	8148 (39.3)	936 (38.5)	258 (34.1)	255 (34.1)	55 (32.0)
Unknown	94 869 (4.9)	663 (3.2)	130 (5.4)	40 (5.3)	39 (5.2)	Suppressed^e^
Health insurance						
Private	637 384 (32.8)	4591 (22.2)	530 (21.8)	180 (23.8)	177 (23.7)	43 (25.0)
Public (Medicare or Medicaid)	1 111 053 (57.1)	14 108 (68.1)	1716 (70.6)	516 (68.3)	510 (68.3)	113 (65.7)
Self-pay, indigent, or charity	109 408 (5.6)	961 (4.6)	79 (3.3)	29 (3.8)	29 (3.9)	Suppressed^e^
Other	87 986 (4.5)	1054 (5.1)	105 (4.3)	31 (4.1)	31 (4.2)	Suppressed^e^
Presence of ≥1 chronic conditions^f^						
Yes	495 959 (25.5)	6047 (29.2)	1659 (68.3)	637 (84.3)	630 (84.3)	Suppressed^e^
No	1 449 872 (74.5)	14 667 (70.8)	771 (31.7)	119 (15.7)	117 (15.7)	Suppressed^e^

Abbreviations: ICU, intensive care unit; NA, not applicable; PHD-SR, Premier Healthcare Database Special COVID-19 Release.

^a^ The source for table data are the PHD-SR (release date, December 9, 2020).

^b^ The total pediatric cohort in the PHD-SR includes all patients 18 years or younger with an emergency department or inpatient encounter from January 1 through October 1, 2020.

^c^ Severe COVID-19 was defined as requiring care in an intensive care or step-down unit, requiring invasive mechanical ventilation, or resulting in death among patients with 1 or more hospitalizations.

^d^ Children of Hispanic or Latino ethnicity were coded as Hispanic or Latino race/ethnicity. Children with known race and either non-Hispanic/Latino ethnicity or unknown ethnicity were coded as non-Hispanic White, non-Hispanic Black, non-Hispanic Asian, or non-Hispanic other race/ethnicity. Children with unknown race and unknown ethnicity were coded as unknown race/ethnicity.

^e^ Cell entries with fewer than 10 patients are suppressed per Premier Inc data use agreements.

^f^ The presence of chronic conditions was assessed using the Agency for Healthcare Research and Quality algorithm. For patients with COVID-19, chronic conditions were assessed using all encounters from January 1, 2019, up to and including the initial COVID-19 encounter. For the total pediatric cohort, which includes patients without COVID-19, chronic conditions were assessed using all encounters from January 1, 2019, up to and including the first encounter from March 1 through October 31, 2020.

Among the cohort of 2430 pediatric patients (11.7%) who were hospitalized with COVID-19, 756 (31.1%) experienced severe COVID-19. An increased association of severe COVID-19 was observed among patients with 1 or more chronic conditions vs those with none (AOR, 3.27; 95% CI, 2.44-4.37); in children aged 2 through 5 years or 6 through 11 years vs those aged 12 through 18 years (AORs, 1.53; 95% CI, 1.11-2.13 and 1.53; 95% CI, 1.04-2.23, respectively); and in male vs female patients (AOR, 1.52; 95% CI, 1.26-1.83) (Figure). There was no statistically significant association between race/ethnicity or insurance type and severe COVID-19.

**Figure.**
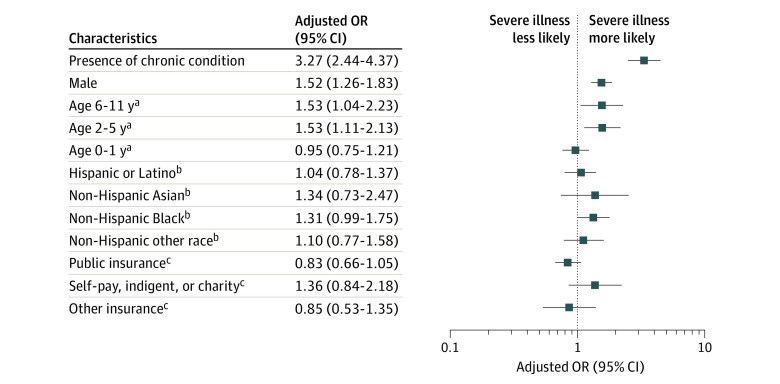
Adjusted Odds Ratios (ORs) of Severe COVID-19 Among Hospitalized Patients 18 Years or Younger An increased association of severe COVID-19 was observed in patients with 1 or more chronic conditions vs those with none, in male vs female patients, and in children aged 2 through 5 years or 6 through 11 years vs children aged 12 through 18 years. An increased association was also found in male vs female patients. Non-Hispanic Black and Hispanic or Latino children with COVID-19 were overrepresented compared with all pediatric patients in the Premier Healthcare Database Special COVID-19 Release. ^a^The reference group is patients aged 12 to 18 years. ^b^The reference group is Non-Hispanic White patients. ^c^The reference group is patients with private insurance.

## Discussion

In this cohort study, nearly one-third (756 [31.1%]) of hospitalized pediatric patients with COVID-19 experienced severe COVID-19, which is consistent with previous findings.^2^ Our analysis revealed an increased association of severe COVID-19 in younger children (those aged 2-11 years) compared with older children (those aged 12-18 years). Although admission to an intensive care unit for younger children may indicate an abundance of caution by clinicians or facility and administrative requirements rather than disease severity, this finding has important clinical and resource planning implications for facilities and clinicians.

Our results suggest that existing chronic conditions and male sex are independently associated with severe COVID-19. Consistent with previous reports, non-Hispanic Black and Hispanic or Latino children with COVID-19 were overrepresented compared with all pediatric patients in the PHD-SR.^5^ We found no statistically significant association between severe disease and race/ethnicity among hospitalized patients when controlling for covariates.

This study has some limitations. First, risk factors associated with severe disease from acute COVID-19 vs multisystem inflammatory syndrome in children cannot be differentiated, as the latter condition does not have its own *ICD-10-CM* diagnosis code. Second, chronic conditions could be underlying, co-occurring, or sequelae of COVID-19 illness. Third, certain chronic conditions might be underdiagnosed in inpatient medical records.^6^ Fourth, we were unable to evaluate associations among infants younger than 12 months.

Although most children with COVID-19 experience mild illness, some children develop serious illness that leads to hospitalization, use of invasive mechanical ventilation, and death. Understanding factors associated with severe COVID-19 disease among children could help inform prevention and control strategies. Reducing infection risk through community mitigation strategies is critical for protecting children from COVID-19 and preventing poor outcomes.
